# Adolescents with Developmental Dyscalculia Do Not Have a Generalized Magnitude Deficit – Processing of Discrete and Continuous Magnitudes

**DOI:** 10.3389/fnhum.2017.00102

**Published:** 2017-03-20

**Authors:** Ursina McCaskey, Michael von Aster, Ruth O’Gorman Tuura, Karin Kucian

**Affiliations:** ^1^Center for MR-Research, University Children’s Hospital ZurichZurich, Switzerland; ^2^Children’s Research Center, University Children’s Hospital ZurichZurich, Switzerland; ^3^Clinic for Child and Adolescent Psychiatry, German Red Cross HospitalsBerlin, Germany; ^4^Neuroscience Center Zurich, University of Zurich and Swiss Federal Institute of Technology ZurichZurich, Switzerland; ^5^Zurich Center for Integrative Human Physiology, University of ZurichZurich, Switzerland

**Keywords:** magnitude processing, visuo-spatial processing, numerosity, adolescents, developmental dyscalculia, functional magnetic resonance imaging

## Abstract

The link between number and space has been discussed in the literature for some time, resulting in the theory that number, space and time might be part of a generalized magnitude system. To date, several behavioral and neuroimaging findings support the notion of a generalized magnitude system, although contradictory results showing a partial overlap or separate magnitude systems are also found. The possible existence of a generalized magnitude processing area leads to the question how individuals with developmental dyscalculia (DD), known for deficits in numerical-arithmetical abilities, process magnitudes. By means of neuropsychological tests and functional magnetic resonance imaging (fMRI) we aimed to examine the relationship between number and space in typical and atypical development. Participants were 16 adolescents with DD (14.1 years) and 14 typically developing (TD) peers (13.8 years). In the fMRI paradigm participants had to perform discrete (arrays of dots) and continuous magnitude (angles) comparisons as well as a mental rotation task. In the neuropsychological tests, adolescents with dyscalculia performed significantly worse in numerical and complex visuo-spatial tasks. However, they showed similar results to TD peers when making discrete and continuous magnitude decisions during the neuropsychological tests and the fMRI paradigm. A conjunction analysis of the fMRI data revealed commonly activated higher order visual (inferior and middle occipital gyrus) and parietal (inferior and superior parietal lobe) magnitude areas for the discrete and continuous magnitude tasks. Moreover, no differences were found when contrasting both magnitude processing conditions, favoring the possibility of a generalized magnitude system. Group comparisons further revealed that dyscalculic subjects showed increased activation in domain general regions, whilst TD peers activate domain specific areas to a greater extent. In conclusion, our results point to the existence of a generalized magnitude system in the occipito-parietal stream in typical development. The detailed investigation of spatial and numerical magnitude abilities in DD reveals that the deficits in number processing and arithmetic cannot be explained with a general magnitude deficiency. Our results further indicate that multiple neuro-cognitive components might contribute to the explanation of DD.

## Introduction

The role of space in numerical processing has been discussed since the very beginning of the numerical and arithmetical scientific history. Galton’s investigation about the spatial orientation of numbers revealed that subjects have internal representations with various visuo-spatial properties (Galton, 1880). Further empirical evidence led to the conclusion that our mental representation of numbers is organized from left to right, nowadays referred to as the mental number line (Dehaene, 1992). Extensively studied psychophysical effects such as the distance effect (subjects are more accurate and faster when comparing numbers that are far apart; e.g., Moyer and Landauer, 1967) and the SNARC-effect (Spatial Numerical Association of Response Codes; subjects respond faster to small numbers with the left hand and to large numbers with the right hand than vice versa; e.g., Dehaene et al., 1993; Schweiter et al., 2005) demonstrate the link to the spatial aspect of numerical processing. Moreover, distance effects were also found for dot patterns, brightness, and length showing that the interaction between space and number also applies for non-symbolic and continuous magnitudes (Moyer and Landauer, 1967; Buckley and Gillman, 1974). Based on this and other empirical evidence, Walsh (2003) proposes in A Theory of Magnitude (ATOM) that time, space and quantity are part of a generalized magnitude system, rather than being processed separately and compared according to their own individual metrics. He argues that this magnitude system is located in the right inferior parietal lobe and evolved from processing visual input in its spatial, quantitative and temporal dimensions in order to produce action-directed motor output (Walsh, 2003; Bueti and Walsh, 2009). This is in line with knowledge of the last decades of imaging studies showing that numerical magnitude processing is conducted mainly in the intraparietal sulcus (IPS) and adjacent regions (e.g., Dehaene et al., 2003; Kaufmann et al., 2013).

Further evidence for the ATOM theory is found in animal studies. Tudusciuc and Nieder (2007, 2009) showed in single-cell experiments with monkeys that continuous (e.g., length) and discrete magnitudes (e.g., arrays of dots) are both processed by shared (fronto-) parietal areas. The underlying visuo-spatial magnitude judgment for length has further been shown to work in a similar way in monkeys and humans (Tudusciuc and Nieder, 2010). In 9-month-old infants a transfer effect across magnitude dimensions of space, number and time could be measured in associative learning, providing support for an innate aspect of the general magnitude system (Lourenco and Longo, 2010). In addition, visuo-spatial abilities are widely reported to be one of the main predictors for later mathematical skills (Assel et al., 2003; Mazzocco and Thompson, 2005; Verdine et al., 2014).

In adults, several behavioral studies report that different magnitude dimensions influence each other. Hurewitz et al. (2006) showed that varying the number, size, or area of dots resulted in a bidirectional interference between the discrete and continuous dimensions of the stimuli. A study from Dormal and Pesenti (2012) found that numerosity and length both affected the processing of time. However, it has been reported that the interference between the different dimensions is not always symmetrical (e.g., Hurewitz et al., 2006). Despite these results, there is also evidence contradicting the theory of a generalized magnitude system. For instance, no correlation among estimations of time, space and numerosities was found in the study by Agrillo et al. (2013).

The ATOM theory was also investigated by means of neuroimaging methods. Functional Magnetic Resonance Imaging (fMRI) studies revealed an activation overlap in the IPS irrespective of the processed magnitude [size/luminance/numbers (Pinel et al., 2004), angles/lines/numbers (Fias et al., 2003), luminance/number line (Vogel et al., 2013), number/space/time (Skagerlund et al., 2016)]. Repetitive transcranial magnetic stimulation (rTMS) over the right IPS induced increased error rates in a length as well as a numerosity estimation task (Dormal et al., 2012). Consistent with these findings, numerosity training coupled with transcranial random noise stimulation (tRNS) led to better and longer lasting improvements in the precision of the approximate number sense. Furthermore, transfer effects to quantity judgments in a time and space task could be found supporting the theory of a shared cognitive and neuronal mechanism (Cappelletti et al., 2013). In a recent fMRI study by Skagerlund et al. (2016), not only IPS, but also insula activation was found across the magnitude dimension time, space and numerosity. This resulted in the suggestion, that both areas are core components of the proposed generalized magnitude system.

In summary, behavioral and neuroimaging studies offer a broad variety of hints about the relationship of space and number. Several findings support the theory of a generalized magnitude system, although contradictory results speaking for a partial overlap or separate magnitude systems are also found (Cappelletti et al., 2014).

In the context of the evidence for and against the existence of a generalized magnitude system, it would be interesting to investigate the relationship between number and space in subjects with specific deficits in number processing, as it is the case in developmental dyscalculia (DD). This disorder is defined as a specific learning disability affecting the acquisition of basic numerical-arithmetical skills that is not explicable on the basis of general mental retardation or of inadequate schooling (WHO, 2010). DD has a prevalence of about 3–7% (Gross-Tsur et al., 1996; Wyschkon et al., 2009) and a persisting character (Shalev et al., 1998, 2005). It can further result in behavioral-emotional problems (Auerbach et al., 2008) and reduced employment opportunities (Parsons and Bynner, 2005). Children with DD show a variety of numerical deficits such as counting, magnitude processing, spatial number representation and fact retrieval (Geary, 1993; Landerl et al., 2004; Rousselle and Noël, 2007; Mussolin et al., 2010b; Landerl, 2013); for an overview see Kucian and von Aster (2015). In addition, domain-general factors such as spatial working memory, executive functions and visuo-spatial abilities are discussed to contribute to the clinical picture of DD (von Aster and Shalev, 2007; Rotzer et al., 2009; Ashkenazi et al., 2013; Fias et al., 2013; Cowan and Powell, 2014; Skagerlund and Träff, 2014). On the neuronal level, there is consistent evidence that children with DD show aberrant activation in the numerical core areas of the parietal brain (e.g., Mussolin et al., 2010a; Kucian et al., 2011a; Ashkenazi et al., 2012). Additionally, a growing number of studies report activation differences in domain-general frontal and occipital areas of the brain (e.g., Davis et al., 2009; Kaufmann et al., 2011; Rosenberg-Lee et al., 2014). In line with this, structural abnormalities along the superior longitudinal fasciculus, connecting the parietal with the frontal brain, have been reported in DD children (e.g., Rykhlevskaia et al., 2009; Kucian et al., 2013; Jolles et al., 2016).

Concerning the visuo-spatial deficits, several behavioral studies investigated the relationship between number and space in children with DD (Landerl et al., 2004, 2009). The study of Rourke and Finlayson (1978) was one of the first to point out that children with DD often have difficulties solving tasks with spatial aspects [e.g., Object Assembly, Block Design of the Wechsler Intelligence Scale for Children (WISC)]. They argue that, “specific deficiencies in arithmetic calculation skills are due to difficulties in visuo-spatial organization and integration” (Rourke and Finlayson, 1978, P. 130). Osmon et al. (2006) found in math-impaired adults that spatial skills (measured with the Judgment of Line Orientation task) differed from math-unimpaired subjects. Similarly, children with DD showed significantly lower performances in all three magnitude-processing abilities proposed by the ATOM theory (number, space, and time) pointing to a deficit in general magnitude processing (Skagerlund and Träff, 2014). On the other hand, Szucs et al. (2013) did not find differences in spatial abilities of dyscalculic children of the same age range. According to their conclusion, deficits in short-term and working memory account for the often reported difficulties in visuo-spatial abilities.

To our knowledge, only one neuroimaging study looked at numerical and spatial abilities in children with DD (Kaufmann et al., 2009). The subjects had either to take a decision about the number of fingers shown or the orientation of the palms. In the spatial condition, children with DD produced significantly stronger activation in the right post-central gyrus/IPS than typically developing (TD) children. Moreover, significant group differences in beta weights could be found in the right inferior parietal lobe (IPL) for the space condition, whilst the number condition produced differences in the left IPL. Kaufmann et al. (2009) concluded that the stronger activation in task relevant regions reflects compensatory mechanism needed in children with DD.

To date, several behavioral studies point to deficiencies in visuo-spatial abilities in dyscalculic children (Rourke and Finlayson, 1978; Landerl et al., 2009; Skagerlund and Träff, 2014). Furthermore, differences in the right IPL, but not left IPL, could be detected in a neuro-imaging study with dyscalculic children for a spatial task (Kaufmann et al., 2009). This contributes to the idea of a shared magnitude system in the right parietal lobe. Hence, not only a deficit in number processing but in the general magnitude system might underlie the mechanisms of DD. However, there are only few studies looking at the relationship of space and number systematically, despite the manifold nature of spatial abilities. Correspondingly, the conducted studies to date have measured simple (e.g., length estimation) as well as complex visuo-spatial abilities (e.g., mental rotation tasks). These tasks involve higher cognitive functions to a different degree and are therefore difficult to compare.

The main goal in our study was to evaluate the theory of a generalized magnitude system by looking at space and number processing using behavioral as well as neuroimaging measures. We aimed to develop a fMRI task that measures discrete quantity processing (non-symbolic numerosity comparison), continuous quantity/visuo-spatial processing (angles comparison) and complex visuo-spatial processing (mental rotation). The second research question intended to examine if there are behavioral and neuronal differences in adolescents with and without DD regarding generalized magnitude processing. We specifically chose to examine adolescents with DD as there is very little literature regarding this age range.

Based on the previous literature, we hypothesize that the numerical as well as the visuo-spatial task containing a magnitude judgment activates a core region for magnitude processing in the IPS (Fias et al., 2003; Walsh, 2003; Pinel et al., 2004; Vogel et al., 2013). Similar behavioral performance and no brain activation differences are predicted to be found between these two conditions in TD adolescents. Secondly, we expect to find deficiencies in behavioral visuo-spatial as well as numerical performance in dyscalculic adolescents (Skagerlund and Träff, 2014). In line with Kaufmann et al. (2009) aberrant activation of parietal regions is anticipated for adolescents with DD. The finding of intact magnitude processing abilities in TD subjects and a deficient performance in DD subjects would point to the existence of a generalized magnitude system. Dissociation between the different magnitude tasks, though, would contradict the ATOM theory.

## Materials and Methods

### Participants and Procedure

Twenty adolescents with DD and 17 TD adolescents between 11.6 and 16.5 years were recruited into this study. Most of the participants were part of a longitudinal project about dyscalculia, 10 participants were additionally recruited for the purpose of this study. The adolescents were either approached in the school setting, School Psychological Services (DD subjects) or applied through our homepage for the participation in the study. Inclusion criteria for all participants were no history of neurological disorders and an Intelligence Quotient (IQ) ≥ 85, measured by the fourth edition of the WISC (Similarities, Block Design, Matrix Reasoning; Petermann and Petermann, 2007). The mathematical performance of adolescents with DD had to be under the cut-off of the standardized numerical test battery BASIS-MATH 4–8 (Basic Diagnosis in Mathematics Education for Grades 4–8; Moser Opitz et al., 2010). TD adolescents had to perform above the cut-off of 67 points in the BASIS-MATH 4–8 (range of the TD children: 67–81). According to these criteria, three participants were excluded from the study. For the fMRI analysis, an additional four subjects were excluded because of movement artifacts or scanner problems. Hence, subsequent analyses are based on data from 16 adolescents with DD (14.1 years) and 14 TD adolescents (13.8 years). Groups were matched for age, gender, handedness and pubertal status, determined by the Edinburgh Handedness Inventory (Oldfield, 1971) and an adapted version of the Self-administered Rating Scale for Pubertal Development (Carskadon and Acebo, 1993) (**Table 1**). Informed consent was obtained from participants when 16 years of age or older and all parents. The study was approved by the Ethics committee of Zurich, Switzerland based on guidelines from the World Medical Association’s Declaration of Helsinki (WMA, 2002).

**Table 1:** Demographic characteristics and scores of numerical abilities, visuo-spatial abilities, domain general cognitive abilities, memory, attention, and reading.

Behavioral measure	DD (*N* = 16) **M* (*SD*)*	TD (*N* = 14) *M* (*SD*)	Test-statistic	*p*
Age	14.1 (1.2)	13.8 (1.3)	0.705^a^	0.487
Gender m/f	4/12	4/10	0.049^b^	0.999
Handedness l/a/r	2/2/12	1/4/9	1.367^b^	0.515
Pubertal status	2.8 (0.8)	2.8 (0.7)	0.610^c^	0.737
**Numerical abilities**				
BASIS-MATH 4-8	50.8 (11.3)	75.1 (4.2)	-7.971^a^	<0.001
KFT 4-12+R quantity comparison	40.2 (4.6)	53.6 (4.9)	-6.395^a^	<0.001
**Visuo-spatial abilities**				
Length estimation (accuracy)	91.9 (7.6)	92.2 (6.0)	0.567^c^	0.715
Size estimation (accuracy)	98.4 (2.1)	97.4 (3.1)	0.567^c^	0.596
Position estimation (accuracy)	63.0 (10.8)	65.0 (11.0)	-0.485^a^	0.632
KFT 4-12+R paper folding	40.4 (10.5)	52.4 (9.0)	-3.234^a^	0.003
DTVP-A form constancy	71.3 (21.7)	83.6 (14.8)	1.415^c^	0.014
DTVP-A copying	40.0 (25.2)	58.8 (29.3)	-1.887^a^	0.070
**Domain general cognitive abilities (WISC-IV)**				
Block design	97 (14.8)	113 (12.1)	-3.075^a^	0.005
Similarities	104 (7.6)	112 (4.7)	-3.562^a^	0.001
Matrix reasoning	101 (8.5)	113 (11.7)	-3.395^a^	0.002
Estimated general IQ	101 (6.5)	113 (5.7)	-5.421^a^	<0.001
**Memory (BTT, WISC-IV)**				
Visuo-spatial working memory	6.0 (1.8)	7.1 (1.9)	0.833^c^	0.235
Verbal memory span	5.6 (1.3)	5.7 (1.2)	-0.335^a^	0.740
Verbal working memory	4.4 (1.1)	5.1 (1.3)	0.659^c^	0.407
**Attention (TAP)**				
Alertness	48.8 (21.5)	52.0 (15.3)	-0.457^a^	0.651
Go-nogo	40.1 (16.1)	40.1 (15.0)	0.659^c^	0.668
**Reading (SLRT-II)**				
Words	19.5 (24.8)	25.6 (23.7)	15.088^b^	0.213
Pseudowords	23.3 (20.7)	33.0 (29.5)	15.088^b^	0.326


*BASIS-MATH 4–8 = basic diagnostic in mathematics education for grades 4–8, KFT 4–12+R = cognitive abilities test [*T*-score], DTVP-A = developmental test of visual perception – adolescent and adult [PR], WISC = wechsler intelligence scale for children [IQ-Score], BTT = block-tapping-test, TAP = Testbattery for attentional performance [PR], SLRT-II = Salzburg reading and orthography test [PR]. ^a^*t*-Test, ^b^Fisher’s exact test, ^c^Kolmogorov–Smirnov-*Z*-test.*

The adolescents visited us twice at the Center for MR-Research of the University Children’s Hospital Zurich. First they completed a neuropsychological session (duration about 2 h) and then underwent the MRI measurement (duration 45 min).

### Neuropsychological Testing

The order of the neuropsychological tests was varied in to ways to avoid order effects. Half of the participants were randomly assigned to the first, the remainder to the second order version of the tests.

#### Numerical Achievement

Numerical achievement was assessed using the BASIS-MATH 4–8 (Moser Opitz et al., 2010), which is the only German test available up to eighth grade to identify numerical deficiencies. The test battery is composed of three difficulty levels measuring several arithmetical abilities such as counting, decimal system, and mental and written calculation. The BASIS-MATH 4–8 is a criterion-referenced test. Criteria for DD are met if the performance is under a threshold value of 67 points (maximum score 83 points). This is interpreted as not reaching mastery of basic mathematical concepts (see Supplementary Material for detailed information about the concept of the test, norms and examples of items).

Additionally, the subtest Quantity Comparison of the Cognitive Abilities Test (KFT 4-12+R; Heller and Perleth, 2000), a norm-referenced test, was used to assess the subjects’ mathematical performance at a peer level. In each item subjects had to indicate if the two presented quantities were the same, or if quantity 1 or quantity 2 was bigger. The presented quantities were geometrical figures (e.g., one circle versus three semicircles), calculations (e.g., 6 × 8 versus 100–48) or units (e.g., 24 h versus 1 day). Adolescents had 10 min time to solve as many items as possible of increasing difficulty. The test values are reported as *T-*scores. Note that for this subtest, data could not be collected for all participants due to the tight schedule of the neuropsychological testing session (three DD missing, six TD missing) and must therefore be interpreted cautiously.

#### Visuo-Spatial Abilities

Because of the multifarious nature of visuo-spatial abilities, the tasks are subdivided into visuo-perceptive, visuo-cognitive and visuo-constructive tasks (Kerkhoff, 2006).

Firstly, visuo-perceptive abilities include amongst others the perception of position in space, length, and distance discrimination (Kerkhoff, 2006). Based on the Birmingham Object Recognition Battery (BORB; Riddoch and Humphreys, 1993) three computerized subtest were programmed on E-prime (Version 2, Psychology Software Tolls Inc., USA) to assess length, size, and position estimation. In the length estimation task subjects had to decide which of two simultaneously presented lines was longer. Correspondingly, in the size task two presented dots had to be compared regarding their size. Both tasks consisted of 30 items and had three difficulty levels with the ratios of 0.75 (simple), 0.85 (medium), or 0.95 (difficult). In the position estimation task, subjects had to match the position of gaps in two circles and indicate if they are at the same or at different positions. Sixty items were shown, whereby simple items differed by 12°, medium by 8°, and difficult by 4°. All tasks were self-paced and responses were given by the keys q (for left is bigger) and p (for right is bigger) or the mouse buttons (index finger for “yes,” middle finger for “no”). Correct responses were balanced for left and right or “same” and “different,” respectively. The percentage of correctly solved items was quantified.

Secondly, visuo-cognitive abilities include in addition to the mere perception of visual stimuli an operation in space, such as mental rotation or change of perspective (Kerkhoff, 2006). In the Form Constancy task of the Developmental Test of Visual Perception – Adolescent and Adult (DTVP-A; Reynolds et al., 2002), subjects were shown a stimulus figure (e.g., square) and asked to find it twice in a series of six figures. The targeted figure appeared in a different size (e.g., smaller), position (e.g., rotated) and/or shade (e.g., only contour), and could be hidden in a distracting background (e.g., rectangles). The test consists of 19 multiple choice items. The percentile rank (PR) of the correctly solved items was quantified. Additionally, subjects solved the Paper Folding subtest of the KFT 4–12+R (Heller and Perleth, 2000). In this task a square sheet of paper was folded 1–4 times and then perforated. Subjects had to indicate how the paper would look when unfolded. The subtest comes in form of a multiple choice task with five choices. Adolescents had 8 min time to solve as many items as possible of increasing difficulty. The test values are reported as *T-*scores.

Lastly, visuo-constructive skills indicate the ability to combine elements to a whole, such as drawing a geometrical figure or assembling cubes to one figure (Kerkhoff, 2006). In the Copying subtest of the DTVP-A (Reynolds et al., 2002), individuals were shown a simple figure and asked to draw it. Subsequent figures were increasingly complex, eventually becoming three-dimensional. Subjects had to draw 12 figures. Following the detailed scoring guidelines of the test manual, each item was scored with 0–3 points. The total test scores are reported as PR. Additionally, the Block Design subtest of the WISC-IV (Petermann and Petermann, 2007) was performed, where subjects had to build a figure with cubes according to a model. The test values are reported as IQ scores.

Regarding the theory of a generalized magnitude system it is important to note, that visuo-perceptive tasks include magnitude processing (because the tasks contain prosthetic dimensions), whilst visuo-cognitive and visuo-constructive tasks do not.

#### Reading Abilities

The 1-Minute-Reading-Task from the Salzburg Reading and Orthography Test (Moll and Landerl, 2010) assessing word and pseudo word reading fluency was used to estimate the reading performance and control for dyslexia. Two sheets of paper with either 156 words or 156 pseudowords of increasing length and difficulty were presented. Subjects had 1 min per sheet to read as many words as possible. The amount of correctly read items was quantified (reported test values are PRs). Because of lacking test norms in grades 7 and 8, values were obtained by interpolating the norms from the test manual (grade 6) and from Kronschnabel et al. (2013; grade 9).

#### Memory Span and Working Memory

In order to control for memory effects, verbal memory span and working memory were assessed using the subtest Digit Span of the WISC-IV (Petermann and Petermann, 2007). In this task subjects had to repeat an auditorily presented sequence of numerals forward and backward, respectively. The sequences had a length of 2–8 numerals. The longest sequence which was reproduced correctly was quantified (reported test values are raw scores, maximum value 8). Visuo-spatial working memory was measured with the suppression-task of the Block-Tapping-Test (Schellig, 1997; Beblo et al., 2004). The task required subjects to reproduce every second block of a previous presented sequence on a board with nine cubes. The sequences had a length of 3–9 cubes. Three items per sequence were presented. The longest sequence which was reproduced correctly twice was quantified (reported test values are raw scores, maximum value 9).

#### Attention

Levels of attention and inhibition were measured by means of the subtests Alertness and Go-Nogo of the Testbattery for Attentional Performance (TAP; Zimmermann and Fimm, 1993). In the Alertness subtest, subjects had to react as quickly as possible to a target stimulus (intrinsic alertness) which was sometimes preceded by a cue stimulus (phasic arousal). In the Go-Nogo subtest, subjects had to react as quickly as possible to a target stimulus (go condition), but inhibit reactions on a second presented stimulus (nogo condition). For each subject the PR of the median RT was quantified (reported test values are PRs).

#### Behavioral Data Analysis

Behavioral data was statistically analyzed with SPSS (Version 20). To assess group differences parametric *t*-tests for independent samples or non-parametric Kolmogorov–Smirnov-*Z*-test were performed if the assumption of normality was violated. Furthermore, differences in the fMRI task performance were examined with mixed-model analyses of variance (ANOVAs) with group as between-subject factor and experimental condition as within-subject factor. In the cases where the assumption of homogeneity of variance was violated, we adjusted the degrees of freedom using the Welch–Satterthwaite method. Effect sizes are reported as Cohen’s *d* for *t*-tests and partial η^2^ for the mixed-model ANOVAs. As suggested by Cohen (1988) effect sizes are interpreted as small (*d* = 0.2, η^2^ = 0.01), medium (*d* = 0.5, η^2^ = 0.06), or large (*d* = 0.8, η^2^ = 0.14).

### Brain Imaging

#### fMRI Paradigm

##### fMRI paradigm design

The fMRI paradigm was newly designed for this study and consist of three experimental and one control condition. In order to avoid strong engagement of executive functions, needed if switching between the four tasks, a block design was chosen rather than an event-related design. Because we aimed to have an optimal signal in terms of high pass filtering (see also Henson, 2007), we designed a paradigm with three runs. Each run lasted 6 min 10 s and consisted of four blocks of one of the experimental conditions alternating with four blocks of the control condition. Order of runs and blocks were counter-balanced between subjects. At the beginning of each block an instruction was shown for 3 s, followed by a blank screen of 500 ms and a block of the experimental or control condition lasting for 30 s. Between the blocks a 13 s rest period with a fixation cross was presented, resulting in a total block length of 46.5 s. The paradigm was self-paced. Nonetheless, stimuli were displayed maximally for 2.5 s with an inter-trial-interval jittered between 1300 and 4300 ms (*M* = 2500 ms).

##### fMRI paradigm task and stimuli

**FIGURE 1:**
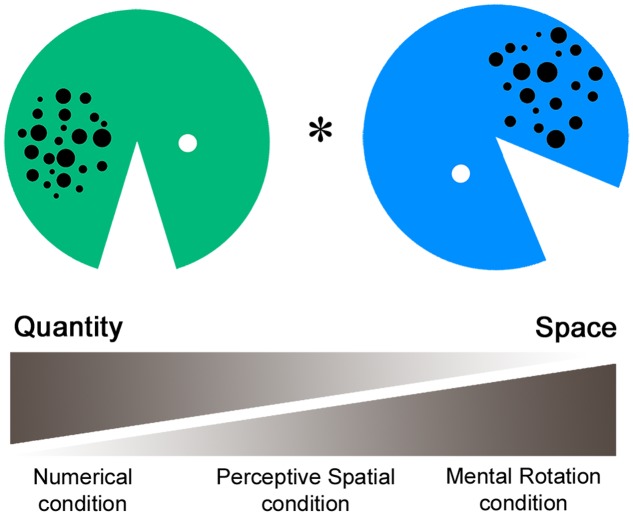
**Functional magnetic resonance imaging paradigm.** In the fMRI paradigm a green and a blue Pacman with varying arrays of dots, mouth size, and rotation angles were presented simultaneously. In the Numerical condition participants had to indicate which Pacman holds more dots, requesting a decision about discrete quantity. Secondly, in the Perceptive Spatial condition participants had to indicate which mouth was bigger, requesting a continuous magnitude and spatial decision. Thirdly, in the Mental Rotation condition participants were asked if the Pacman would face toward each other when rotating to an upright position, requesting a mainly spatial decision. Finally, in the control condition subjects had to indicate which Pacman was green.

The fMRI paradigm intends to measure perceptive and cognitive spatial as well as magnitude processing. In the task a green and a blue Pacman with varying arrays of dots, mouth size, and rotation angles were presented simultaneously (**Figure 1**). In the first experimental condition (Numerical condition), participants had to compare the dot arrays and indicate which Pacman holds more dots in his belly. This non-symbolic magnitude comparison task requests a decision about a discrete quantity. Secondly, in the Perceptive Spatial condition subjects were asked which Pacman’s mouth was bigger. This task requires a visuo-perceptive and continuous magnitude decision. Thirdly, in the Mental Rotation condition adolescents were asked to judge if the Pacman would face toward each other if rotated to an upright position. This task intends to measure visuo-spatial ability, which is not intertwined with a magnitude decision. Additionally, it involves higher order cognitive functions (executive functions), thereby representing a more complex spatial task than the Perceptive Spatial and Numerical conditions. Finally, the control task is a simple color discrimination task including no judgment of magnitude or visuo-spatial abilities.

A single stimulus consisted of a Pacman with a diameter of 13.2 cm created in Adobe Photoshop. The dot arrays were controlled for dot size, total surface and density. Dots varied between 0.25 and 1 cm in diameter, had a total surface of 5.9 cm^2^ and were either spread on a small (5 × 6 cm) or al large area (6 × 7 cm; see also Gebuis and Reynvoet, 2012). Dot arrays contained between 14 and 28 dots, representing three ratios of varying difficulty (reference array for comparison = 20 dots, simple: ratio = 0.70, 14, or 28 dots; medium: 0.83, 17, or 24 dots; and difficult: 0.91, 18, or 22 dots). Similarly, for the comparison of the mouth angles three difficulty levels were set according to the ratio of the angles (reference angle = 45°, simple: ratio = 0.76, 34°, or 59°; medium: 0.93, 41.5°, or 48°; and difficult: 0.97, 43°, or 46°). In the Mental Rotation task 135° (simple), 180° (medium), or 225° (difficult) of total rotation had to be completed. Items were set at six different starting positions (45°, 90°, 135°, 225°, 270°, and 315°) relative to the upright position (0°). Finally, colors for the control task were of the same luminance to avoid any comparative processing of brightness (see also Pinel et al., 2004). The ratios of this task were set carefully based on a pilot behavioral study with 60 children (mean age 12.9 years) and a subsequent testing phase in the scanner with adults (for results see Grond et al., 2012). Critically, as the stimuli were exactly the same over all four tasks, conditions are highly comparable in terms of visual input, eye movements and motor responses. The stimuli were presented in pairs horizontally aligned via a video goggles system (VisuaStimDigital, Resonance Technology Inc., USA). The distance from the screen was therefore the same for all subjects (30° in the horizontal visual field of view). The subjects answered by a button press of the dominant hand (index finger for “yes” or “left,” middle finger for “no” or “right”), which was recorded using an MRI compatible response box (Lumina Respond Pad, Cedrus Corporation, USA). The paradigm was programmed on E-Prime (Version 2, Psychology Software Tolls Inc., USA). Stimuli were pseudo-randomized and correct answers balanced for left/right.

#### Image Acquisition

Magnetic resonance imaging data were acquired on a 3T General Electric Discovery 750 Scanner (GE Medical Systems, USA) using an 8-channel head coil. Whole brain functional images were acquired sequentially with a gradient echo EPI sequence [38 slices, 3 mm slice thickness (ST), 0.3 mm interslice gap, 64 × 64 matrix size (MS), field of view (FOV) = 240 mm, flip angle (FA) = 74°, echo time (TE) = 32 ms, repetition time (TR) = 1900 ms]. Additionally, a T1-weighted structural image was obtained with a spoiled gradient echo sequence (3D SPGR, ST = 1 mm, no interslice gap, MS = 256 × 256, FOV = 256 mm, FA = 8°, TE = 5 ms, TR = 11 ms).

Participants were carefully instructed and supplied with hearing protection before entering the scanner. To minimize head motion, the head was stabilized with padding.

#### fMRI Preprocessing

The fMRI data were analyzed by means of Statistical Parametric Mapping (SPM8, Wellcome Trust Centre for NeuroImaging, UK) running under Matlab (Release 2012b, The MathWorks Inc., USA).

Three dummy scans, acquired to stabilize magnetization at the beginning of the scan, were excluded from the analysis. Afterward, the subjects’ functional scans were realigned with rigid body transformations using the mean image as a reference scan. Six motion parameters (translation in *x*, *y*, and *z* direction as well as rotation in pitch, roll and yaw) were stored and included later in the analysis to control for motion. The mean functional image was then coregistered to the subjects’ T1-weighted anatomical scan. In a next step, the individual anatomical scan was segmented into gray and white matter according to tissue probability maps of a pediatric atlas (NIH Pediatric Database; Fonov et al., 2009, 2011). The pediatric atlas, created for the ages 4.5–18.5 years, was chosen since it provided better segmentation results than the adult template. Parameters from the coregistration and segmentation were then applied to the functional scans to normalize images into the Montreal Neurological Institute (MNI) space. Finally, the functional images were smoothed with a Gaussian kernel of 6 mm FWHM (full width half maximum).

#### fMRI Statistics

The first level analysis was performed using a mass-univariate approach based on the GLM (General Linear Model). The time series from each subject were modeled with an event related design for the experimental and control condition using a canonical HRF (hemodynamic response function). The six subjects’ motion parameters were entered as additional regressors. Slow signal drifts and serial correlations were accounted for by using a high-pass filter of 128 s and a first level autoregressive model during maximum-likelihood estimation of the GLM parameters.

At the second level, a full factorial analysis with the factors group (DD, TD) and task (Numerical, Perceptive Spatial, Mental Rotation) was conducted for the contrast images experimental > control condition.

Statistical results are shown with a threshold of *p* < 0.05 family-wise error (FWE) correction and minimum cluster size of *k* ≥ 5 voxels. Alternatively results are presented with a less strict significance level of *p* < 0.001, corrected for multiple comparisons using a cluster-extent threshold of *k* ≥ 23 voxels (621 mm^3^). According to Slotnick (2008), the spatial autocorrelation of the data was estimated. Then a Monte Carlo simulation was run with 10’000 iterations, using a type I error voxel activation probability of 0.001, and an estimated FWHM as a Gaussian smoothing kernel in order to derive the cluster extent threshold yielding the desired correction for multiple comparisons at a *p* < 0.05 level (Slotnick, 2004).

Anatomical localization of the fMRI results was attained through the SPM Anatomy Toolbox v2.0 (Eickhoff et al., 2005, 2007) and is reported in the MNI coordinate space.

## Results

### Behavioral Data

The neuropsychological results and the demographic data for all participants are summarized in **Table 1**. All participants reached normal range of intelligence in the WISC-IV (DD IQ = 92–117, TD IQ = 97–122). However, group differences could be detected in the estimated general IQ (*p* < 0.001, *d* = 1.98) and the single subtests (*p* ≤ 0.01; **Table 1**). Differences in IQ scores between a group of children with learning disabilities and a control group are often reported in the literature (Geary et al., 2000; Landerl, 2013; Willcutt et al., 2013). One reason for this is that IQ tests are not independent from numerical skills. Furthermore, the fact that we did not have artificially matched IQ groups, allowed us to include all DD subjects, resulting in a sample that represents the clinical population of persons with DD well. Importantly, none of our DD adolescents performed below an IQ of 92, meeting the criteria according to the ICD-10 (WHO, 2010) and DSM-V (IQ > 70, ± 5 point measurement error; American Psychiatric Association, 2013). The IQ was not entered as a covariate in the subsequent behavioral and fMRI analysis, since IQ is not independent from the effects of interest (Miller and Chapman, 2001; Dennis et al., 2009; Field, 2009).

Regarding the comorbid disorders such as attention deficit and hyperactivity disorder, dyslexia and working memory deficits, groups did not differ significantly in any measurement of attention, reading or memory performance (all *p* ≥ 0.21; **Table 1**).

#### Numerical Achievement

Numerical abilities, assessed by the Basis-Math, differed highly between the TD and the DD group (*p* < 0.001, *d* = 2.8; **Table 1**). Importantly, subjects with DD performed consistently worse in all three difficulty levels, showing deficits even in very basic arithmetical skills [level 1: DD *M* = 37.7, *SD* = 8.5, TD *M* = 50.2, *SD* = 1.7, *t*(16.4) = -5.76, *p* < 0.001, *d* = 2.01, level 2: DD *M* = 8.5, *SD* = 3.3, TD *M* = 14.4, *SD* = 1.7, *t*(23.2) = -6.15, *p* < 0.001, *d* = 2.19, level 3: DD *M* = 4.6, *SD* = 2.4, TD *M* = 10.5, *SD* = 2.4, *t*(28) = -6.69, *p* < 0.001, *d* = 2.45]. Similarly, the groups differed in the curricular test Quantity Comparison (KFT 4–12+R), with the DD subjects scoring significantly lower than the matched TD group (*p* < 0.001, *d* = 2.86).

#### Visuo-Spatial Abilities

In the visuo-perceptive tasks, accuracy was measured by calculating the ratio of the correctly solved items compared to the total number of items. The results revealed that both groups were able to solve the length and size estimation task well. The position estimation task was more difficult for the adolescents as seen by the lower accuracy values. All participants solved the task with an accuracy level of over 50%, except for two DD and two TD subjects. However, no significant differences could be found between the groups in any of the visuo-perceptive tasks (all *p* ≥ 0.05; **Table 1**). Regarding the measured reaction times (RT), both groups solved the task with a similar speed [length estimation: DD *M* = 1022, *SD* = 512, TD *M* = 895, *SD* = 228, *t*(17.9) = 0.85, *p* = 0.408, *d* = 0.78, size estimation: DD *M* = 848, *SD* = 283, TD *M* = 777, *SD* = 203, *t*(26) = 0.77, *p* = 0.449, *d* = 0.29, position estimation: DD *M* = 1634, *SD* = 620, TD *M* = 1716, *SD* = 470, *t*(26) = -0.40, *p* = 0.696, *d* = 0.15].

In the visuo-cognitive abilities, significant differences between groups could be found in both tests. Adolescents with DD performed worse than the TD adolescents in the Form Constancy subtest of the DTVP-A (*p* < 0.05, *d* = 0.65). The DD group also scored significantly lower in the Paper Folding subtest of the KFT 4–12+R compared to the TD group (*p* < 0.01, *d* = 1.22; **Table 1**).

Finally, in the visuo-constructive tasks, no significant difference in performance was observed in the Copying subtest of the DTVP-A, although, a trend-level difference in performance was detected, with dyscalculic adolescents reaching a mean PR of 40, whereby TD adolescents score at PR 59 (*p* = 0.07, *d* = 0.69). In the Block Design subtest of the WISC-IV, subjects with DD performed significantly worse than TD subjects (*p* < 0.01, *d* = 1.13; **Table 1**).

#### Behavioral Results of the fMRI Task

As the fMRI paradigm was newly designed for this study and adolescents solved it in a self-paced mode, we first looked at some general features of the paradigm before looking at group differences. Hence, the number of solved items was quantified and entered into a mixed-model ANOVA with experimental condition as a within-subject factor and group as a between-subject factor. Results showed a significant effect of condition [*F*(2.4,67.4) = 85.5, *p* < 0.001, η^2^ = 0.75] and no effect of group [*F*(1,28) = 0.001, *p* = 0.976, η^2^ = 0.001] or interaction [*F*(2.4,67.5) = 0.09, *p* = 0.942, η^2^ = 0.003]. *Post hoc* tests revealed that more items were solved in the control condition (*M* = 38.5, *SD* = 1.3) compared to the Numerical (*M* = 33.0, *SD* = 2.5), Perceptive Spatial (*M* = 33.9, *SD* = 2.4), as well as the Mental Rotation condition (*M* = 32.0, *SD* = 3.1; all *p* < 0.001). Furthermore, the number of solved items in the Perceptive Spatial condition was slightly higher compared to the Mental Rotation condition (*p* = 0.002). However, in all three experimental conditions subjects solved in average between 32 and 34 items. We therefore conclude that the tasks are comparable between experimental conditions and groups.

**FIGURE 2:**
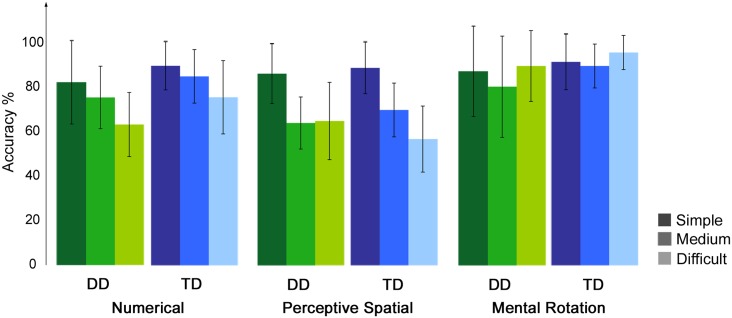
**Behavioral results of the fMRI paradigm.** Accuracies of the fMRI paradigm for each experimental condition split up for the three difficulty levels, the lighter the colors the more difficult the tasks.

Accuracy and RT were calculated for each condition, excluding trials in which RT was smaller than 300 ms and misses (**Table 2**). For the control condition a single value was calculated over the three runs. Correct and incorrect trials were included in the subsequent analysis. All participants performed in the three runs above chance level (50%). Regarding accuracy, an ANOVA with experimental condition and difficulty level as a within-subject factors and group as a between-subject factor revealed significant effects of condition [*F*(1.8,50.0) = 24.32, *p* < 0.001, η^2^ = 0.47] and difficulty [*F*(1.6,43.7) = 24.51, *p* < 0.001, η^2^ = 0.47]. Furthermore, the interaction condition by difficulty level reached significance [*F*(3.4,96.4) = 16.06, *p* < 0.001, η^2^ = 0.37]. No effect of group or further interaction was significant (all *p* ≥ 0.07). With increasing difficulty level the accuracy decreases constantly in the Numerical as well as in the Perceptive Spatial condition, showing that the task was feasible for both groups and the set levels were perceived as increasingly difficult in both magnitude processing conditions (**Figure 2**). In the Mental Rotation condition, however, accuracy was similar for all difficulty levels (**Figure 2**). This might be explained by different strategies used to solve the task. *Post hoc* tests showed that participants scored higher (*p* < 0.01) in the Mental Rotation (*M* = 0.90, *SD* = 0.15) compared to the Numerical (*M* = 0.79, *SD* = 0.10) and the Perceptive Spatial condition (*M* = 0.71, *SD* = 0.09), and in the Numerical compared to the Spatial Perceptive condition (*p* < 0.01; **Table 2**). Regarding difficulty, more items were solved correctly (*p* < 0.001) of the simple (*M* = 0.88, *SD* = 0.12) compared to the medium level (*M* = 0.78, *SD* = 0.10). However, the accuracies of the difficult level (*M* = 0.75, *SD* = 0.10) did not significantly differ from the medium level (*p* = 0.335; **Figure 2**).

**Table 2:** Mean accuracies and RT of the fMRI paradigm conditions for DD and TD adolescents as well as the total mean.

Behavioral measure	DD (*N* = 16)	TD (*N* = 14)	Total
	*M (SD)*	*M (SD)*	*M (SD)*
**fMRI Paradigm accuracy [%]**			
Numerical condition	73.9 (9.1)	84.1 (9.1)	78.7 (10.3)
Perceptive Spatial condition	70.9 (9.9)	71.2 (8.9)	71.0 (9.3)
Mental Rotation condition	86.4 (18.4)	93.1 (7.6)	98.5 (14.6)
Control condition	98.0 (2.6)	99.0 (1.5)	98.0 (2.2)
**fMRI Paradigm RT [ms]**			
Numerical condition	1190 (350)	1239 (267)	1213 (299)
Perceptive Spatial condition	1320 (254)	1313 (313)	1316 (282)
Mental Rotation condition	1312 (383)	1333 (361)	1322 (367)
Control condition	571 (124)	582 (91)	577 (106)


The analysis of the RT revealed a significant effect of condition [*F*(2.4,65.9) = 103.48, *p* < 0.001, η^2^ = 0.79]. Participants were significantly faster when solving trials from the control condition (*M* = 577, *SD* = 106), compared to the three experimental conditions (Numerical: *M* = 1213, *SD* = 299, Perceptive Spatial: *M* = 1316, *SD* = 282, Mental Rotation: *M* = 1322, *SD* = 367; **Table 2**). RT were also lower for the Numerical compared to the Perceptive Spatial condition (*p* = 0.010). No effect of group or further interaction was significant (all *p* ≥ 0.85).

Motion determined by the total displacement of the motion fingerprint (Wilke, 2012) was not significantly different between runs, experimental conditions or groups (all *p* ≥ 0.14).

### fMRI

#### fMRI Task Effects

**FIGURE 3:**
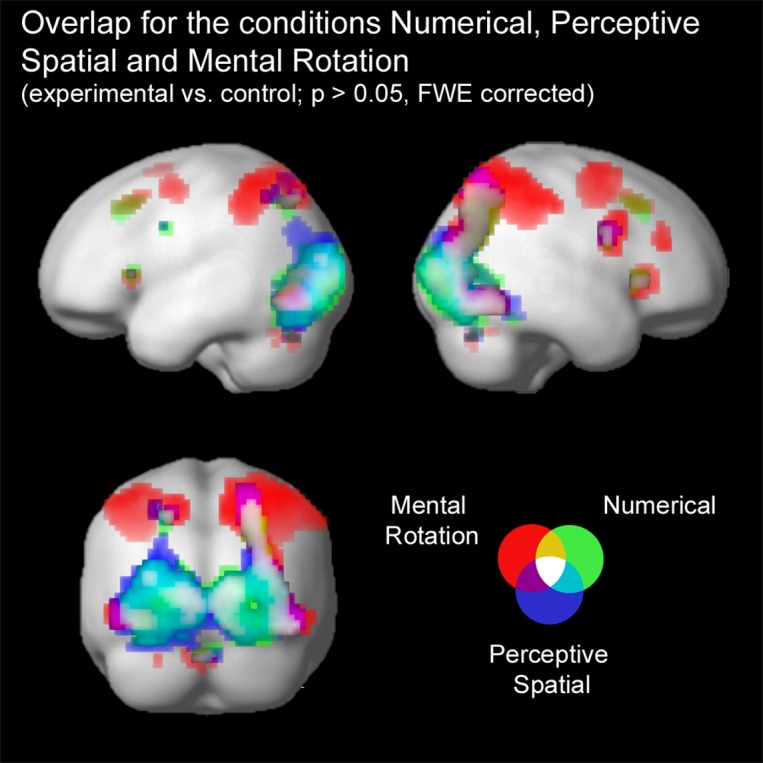
**Brain activation of the three experimental conditions: Numerical (green), Perceptive Spatial (blue), and Mental Rotation (red).** Overlap between the magnitude conditions (Numerical and Perceptive Spatial) is shown in turquoise, between the space conditions (Perceptive Spatial and Mental Rotation) in violet, and between the Mental Rotation and the Numerical condition in yellow. Commonly activated regions in all three experimental tasks appear in white. Note that the overlap corresponds to the results of the conjunction analyses.

Firstly, conjunction analyses were conducted to examine jointly used regions over all experimental conditions (experimental > control condition; FWE corrected at *p* < 0.05; **Figure 3** and **Table 3**). A conjunction analysis of the three experimental tasks over both groups activated mainly regions in the bilateral middle (MOG) and inferior occipital gyrus (IOG) extending into the right inferior and superior parietal lobe (SPL; see white colored areas of **Figure 3** and Supplementary Figure S1). Further activation could be found in the right calcarine gyrus, right insula and the left SPL. A similar pattern was detected (turquoise and white colored areas of **Figure 3** and Supplementary Figure S2) using a conjunction analysis only with the tasks containing a magnitude decision (Numerical condition and Perceptive Spatial condition). However, the activation in the visual areas (MOG, IOG) is more pronounced and additional activation in the cerebellum (vermis) is found. The activation pattern for the conjunction analysis only with the tasks containing a spatial decision (Perceptive Spatial condition and Mental Rotation condition) revealed solely an additional activation in the right precentral gyrus (violet and white colored areas of **Figure 3** and Supplementary Figure S3).

**Table 3:** Brain areas that showed significant activation in the conjunction analyses for all experimental conditions, magnitude conditions and visuo-spatial conditions, respectively (*p* < 0.05, *k* ≥ 5, FWE corrected).

Region	Cluster size	Peak *t*-value	Peak MNI coordinates		
			
			*x*	*y*	*z*
**Conjunction analyses**					
*Numerical, Perceptive Spatial and Mental Rotation (experimental conditions)*					
*([Numerical > Control] ∩ [Perceptive Spatial > Control] ∩ [Mental Rotation > Control])*					
R middle occipital gyrus	249	6.59	28	-71	33
R inferior occipital gyrus (assigned to fusiform gyrus)		6.57	43	-65	-12
R calcarine gyrus (assigned to V1)	144	7.79	13	-86	6
N/A (assigned to V1)	119	6.80	-14	-86	3
L inferior occipital gyrus	38	6.34	-35	-77	-9
R insula	10	5.27	34	22	3
L superior parietal lobe (assigned to area 7A)	6	5.21	-20	-71	48
L middle occipital gyrus	6	5.42	-29	-89	15
*Numerical, Perceptive Spatial (magnitude conditions)*					
*([Numerical > Control] ∩ [Perceptive Spatial > Control])*					
L middle occipital gyrus	1850	9.60	-29	-89	15
R middle occipital gyrus		8.03	31	-86	15
R calcarine gyrus (assigned to V1)		7.79	13	-86	6
R insula	10	5.27	34	22	3
L superior parietal lobe (assigned to area 7A)	9	5.21	-20	-71	48
Cerebellar vermis	7	5.22	4	-71	-27
*Perceptive Spatial, Mental Rotation (visuo-spatial conditions)*					
*([Perceptive Spatial > Control] ∩ [Mental Rotation > Control])*					
R inferior temporal gyrus (assigned to fusiform gyrus)	402	7.24	46	-68	-12
R superior parietal lobe (assigned to area 7A)		6.74	22	-68	57
R calcarine gyrus (assigned to V1)	147	8.09	13	-89	6
N/A (assigned to V1)	119	6.80	-14	-86	3
L inferior occipital gyrus	87	6.34	-35	-77	-9
R precentral gyrus	31	5.90	49	4	30
L superior parietal lobe (assigned to area 7A)	12	5.21	-20	-71	48
R insula	10	5.27	34	22	3
L inferior parietal lobe (assigned to intraparietal sulcus)	8	5.06	-29	-56	48
L middle occipital gyrus	6	5.42	-29	-86	15


**FIGURE 4:**
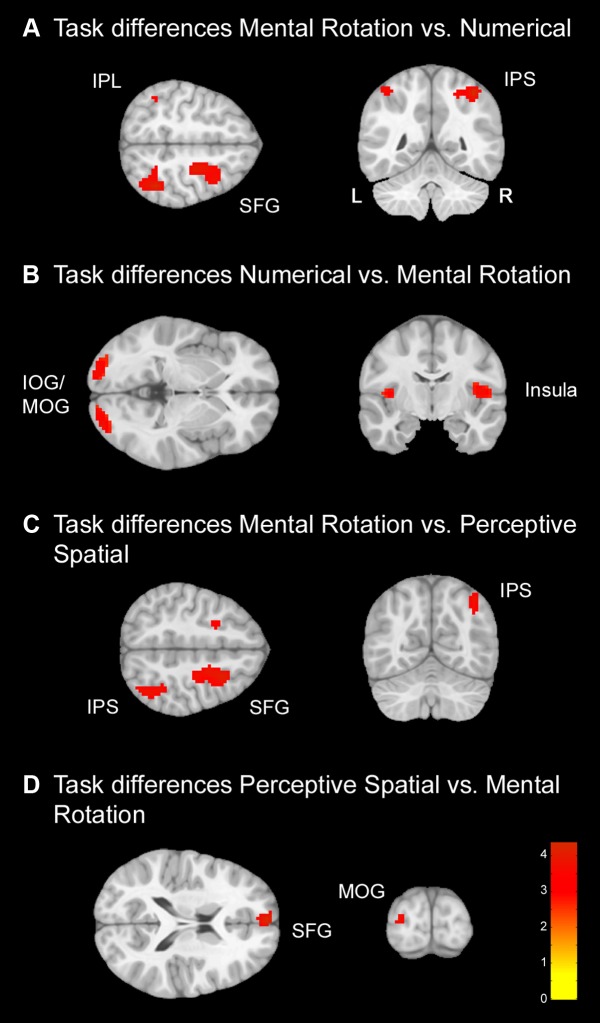
**Task differences for typically developing (TD) adolescents.** Task differences shown on a pediatric template (Fonov et al., 2009) with a significance level of *p* < 0.001, cluster extend corrected. **(A)** Increased activation for the Mental Rotation versus Numerical condition. **(B)** Increased activation for the Numerical versus Mental Rotation condition. **(C)** Increased activation for the Mental Rotation versus Perceptive Spatial condition. **(D)** Increased activation for the Perceptive Spatial versus Mental Rotation condition. IOG, inferior occipital gyrus; IPL, inferior parietal lobe; IPS, intraparietal sulcus; MOG, middle occipital gyrus; SFG, superior frontal gyrus.

Secondly, comparisons between the conditions were conducted to examine regions used specifically for the single conditions (**Figure 4** and **Table 4**). In contrast to the conjunction analysis only data from the TD group was used in this analysis in order to avoid any influence of the DD group on the results. Several regions survived FWE correction, but results are reported with a *p* < 0.001 significance level as described before.

**Table 4:** Brain areas that showed significant activation for the different task comparisons in typically developing adolescents (*p* < 0.001, *k* ≥ 23, cluster-extend corrected).

Region	Cluster size	Peak *t*-value	Peak MNI coordinates		
			
			*x*	*y*	*z*
**Task effects**					
*Numerical >Perceptive Spatial*	*n.s.*				
*Perceptive Spatial > Numerical*	*n.s.*				
*Mental Rotation > Numerical*					
N/A (adjacent to the R superior frontal gyrus)	301	4.33	31	1	45
R superior frontal gyrus		4.29	25	-8	60
R inferior parietal lobe (assigned to intraparietal sulcus)	217	4.70	40	-50	54
R superior parietal lobe (assigned to intraparietal sulcus)		3.94	28	-56	63
R middle occipital gyrus	29	3.87	40	-74	30
L inferior parietal lobe	28	4.27	-44	-56	60
*Numerical > Mental Rotation*					
L superior frontal gyrus (assigned to frontal pole)	215	4.87	-14	64	21
L superior medial gyrus		4.07	-2	61	18
R inferior occipital gyrus (assigned to V3)	213	6.05	22	-92	-6
R middle occipital gyrus		5.22	31	-92	3
R insula/rolandic operculum	129	5.13	49	-5	9
L calcarine gyrus	129	5.03	-17	-98	-3
L insula	36	4.32	-41	-11	9
*Mental Rotation > Perceptive Spatial*					
R superior frontal gyrus	405	4.79	22	1	66
R middle frontal gyrus		4.52	25	10	45
R inferior parietal lobe (assigned to intraparietal sulcus)	146	4.05	40	-50	54
L middle frontal gyrus	44	3.71	-26	-2	54
R supramarginal gyrus (assigned to inferior parietal lobe)	40	3.96	58	-29	45
R middle occipital gyrus	26	4.44	40	-71	30
*Perceptive Spatial > Mental Rotation*					
L superior frontal gyrus (assigned to frontal pole)	119	4.43	-5	61	18
L middle occipital gyrus (assigned to V3)	27	3.76	-29	-95	12
L middle temporal gyrus	24	4.49	-62	-35	0


Numerical versus Perceptive Spatial: The contrast Numerical versus Perceptive Spatial condition revealed no significant differences.

Perceptive Spatial versus Numerical: The contrast Perceptive Spatial versus Numerical condition revealed no significant differences.

Mental Rotation versus Numerical: The Mental Rotation task elicited greater activation compared to the Numerical task in the right IPS, the right MOG reaching into IPL, left IPL and the right superior frontal gyrus (SFG) (**Figure 4A**).

Numerical versus Mental Rotation: The opposite contrast revealed higher activation in the bilateral IOG/MOG, bilateral insula, left superior medial gyrus and SFG (**Figure 4B**).

Mental Rotation versus Perceptive Spatial: For the Mental Rotation task activation increase was found in the right IPS (extending into the angular gyrus), the supramarginal gyrus, the MOG, and bilateral SFG/superior medial gyrus compared to the Perceptive Spatial task (**Figure 4C**).

Perceptive Spatial versus Mental Rotation: The Perceptive Spatial condition, however, revealed higher activation in the left MOG, the medial temporal gyrus and the SFG (**Figure 4D**).

#### fMRI Group Differences

**FIGURE 5:**
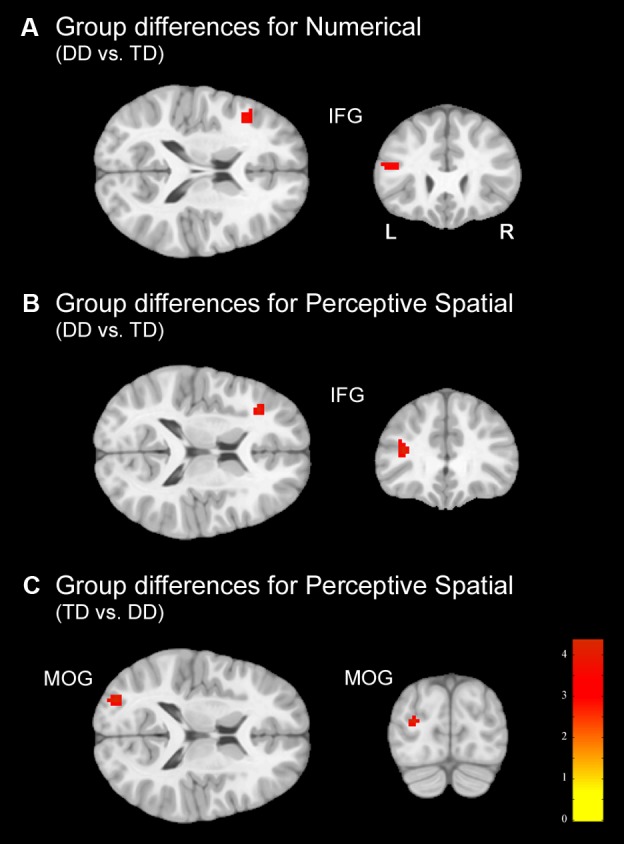
**Group differences.** Group differences shown on a pediatric template (Fonov et al., 2009) with a significance level of *p* < 0.001, cluster extend corrected. **(A)** Increased activation in the dyscalculic adolescents compared to the TD group for the Numerical condition. **(B)** Increased activation in the adolescents with developmental dyscalculia compared to the TD peers for the Perceptive Spatial condition. **(C)** Increased activation in TD adolescents compared to dyscalculic adolescents for the Perceptive Spatial condition. IFG, inferior frontal gyrus; MOG, middle occipital gyrus.

In the Numerical condition, adolescents with DD showed increased activation in the left inferior frontal gyrus (IFG; pars triangularis) compared to TD adolescents (**Figure 5A** and **Table 5**). TD subjects did not elicit higher activation in any regions compared to dyscalculic subjects in the Numerical task.

**Table 5:** Brain areas that showed significant activation in the different conditions when contrasting DD adolescents and TD adolescents (*p* < 0.001, *k* ≥ 23, cluster-extend corrected).

Region	Cluster size	Peak *t*-value	Peak MNI coordinates		
			
			*x*	*y*	*z*
**Task effects**					
*Numerical DD > TD*					
L inferior frontal gyrus	37	4.26	-41	19	21
*Numerical TD > DD*	*n.s.*				
*Perceptive Spatial DD > TD*					
L inferior frontal gyrus	27	4.35	-35	31	15
*Perceptive Spatial TD > DD*					
L middle occipital gyrus	24	4.12	-29	-86	15
*Mental Rotation DD > TD*	*n.s.*				
*Mental Rotation DD > TD*	*n.s.*				


In the Perceptive Spatial condition dyscalculic adolescents showed increased activation in the left IFG (pars triangularis), whereas TD adolescents had higher activation in the left MOG (**Figures 5B,C** and **Table 5**).

Finally, in the Mental Rotation task neither the DD nor the TD adolescents showed activation differences when comparing them against each other.

## Discussion

The link between space and number has been discussed and investigated in the literature for some time, leading to the theory that time, space, and number might be part of a generalized magnitude system located in the parietal cortex (Walsh, 2003). In the present study, we therefore examined the relationship of space and number by means of behavioral and neuroimaging methods. The fact that we studied this relationship in TD adolescents as well as in adolescents with number processing deficits (DD) enabled us to investigate the mechanisms of the generalized magnitude system in more detail. The tasks containing continuous and discrete magnitude decisions elicited an almost identical neuronal network in the higher order visual areas and parietal magnitude processing areas, revealing no significant differences in the contrast between these two task conditions. On the other hand, simple visuo-spatial processing differed from Mental Rotation, such that Mental Rotation activated more right parietal and frontal regions and Perceptive Spatial processing activated mainly higher order visual areas. In the second research question, we examined the behavioral and neuronal differences in adolescents with and without DD during spatial and numerical processing. Beside the known deficit in numerical-arithmetical abilities, adolescents with DD showed additional difficulties in the cognitive and constructive visuo-spatial abilities of the behavioral tests. However, DD adolescents seem to have well developed abilities in processing discrete and continuous (perceptive visuo-spatial) magnitudes. Brain imaging results further revealed that DD adolescents engage more domain-general frontal regions when solving magnitude tasks, whilst TD adolescents activate task-specific areas to a higher extent.

### A Generalized Magnitude System in the Occipito-Parietal Lobe

The main goal of this study was to elaborate the theory of a generalized magnitude system looking at number and visuo-spatial processing. In our fMRI task, we therefore aimed to measure discrete quantity processing (Numerical condition), continuous quantity/simple visuo-spatial processing (Perceptive Spatial condition) and complex visuo-spatial processing, the latter without an explicit magnitude decision (Mental Rotation).

At first, the neuronal and behavioral analyses of the tasks containing magnitude processing (prosthetic dimensions) were of interest. The conjunction analysis with the tasks involving magnitude processing revealed a network with a prominent activation cluster in the occipital and the IPL/SPL (**Figure 3**). Moreover, the task-specific comparisons between the Perceptive Spatial and the Numerical condition did not lead to any significant differences. The activation in the right IPL and SPL is in line with several studies looking at different modalities of magnitude. A study with numerosity and length processing elicited activation in the bilateral IPS (IPL/SPL; Dormal and Pesenti, 2009). The right IPS and posterior SPL were found to be commonly involved when performing number line and brightness estimations (Vogel et al., 2013). Finally, the study of Pinel et al. (2004) looking at number, size and luminance comparisons revealed activation in bilateral anterior IPS. Furthermore, our results are in agreement with the findings of brain stimulation studies showing that disrupting the right IPS leads to increased error rates in numerosity as well as length estimation (Dormal et al., 2012) and stimulating the right parietal lobe improved numerosity but also quantity judgments in time and space (Cappelletti et al., 2013).

Additional clusters of activation were revealed in the bilateral higher order visual areas MOG/IOG as well as in the visual cortex (V1). This was not the case in the aforementioned studies (Dormal and Pesenti, 2009; Vogel et al., 2013). However, studies looking at spatial as well as non-symbolic numerosity and temporal processing, respectively, reported activation in the same areas (Kaufmann et al., 2008; Gijssels et al., 2013). Furthermore, Roggeman et al. (2011) disentangled in an adaptation study different stages of non-symbolic quantity processing. When looking at large versus small processed numerosities they obtained similar activations in the bilateral MOG (Roggeman et al., 2011). Therefore, it seems that a rough estimation of quantity may already be performed in the occipital lobe. Our results might extend these findings by showing that not only numerosities but also continuous magnitudes are processed in this approximate occipito-parietal stream, since the Mental Rotation task, which did not require any magnitude estimation, did not induce occipital activation to the same extent.

Regarding the behavioral data, the adolescents performed better in the Numerical task than the Perceptive Spatial task. However, previous results showed that comparison of continuous stimuli (Perceptive Spatial task) is easier than discrete magnitude comparisons (Numerical task; Leibovich and Henik, 2014). This finding was corroborated by the results of our pilot study (Grond et al., 2012) and Kucian et al. (2016) who used the same paradigm as in the present study, but with identical ratios between the conditions. Therefore, the difficulty of the Perceptive Spatial task was augmented in the present study by using higher ratios with the aim to reach similar performance levels for the magnitude conditions. As a result thereof, the better performance in the Numerical task can partially be explained by the adapted ratios. It should further be considered that due to the different ratios, the difficulty of the conditions is not directly comparable.

To summarize, our findings about magnitude processing argue for a common network irrespective of whether discrete or continuous magnitude judgment is used, in agreement with several studies (Fias et al., 2003; Hurewitz et al., 2006; Tudusciuc and Nieder, 2007, 2009; Dormal and Pesenti, 2009; Agrillo et al., 2011) and the proposed generalized magnitude system of Walsh (2003).

In a next step, comparisons between the magnitude and visuo-spatial conditions were drawn. Not surprisingly, task specific differences between the Mental Rotation task and the Perceptive Spatial or Numerical tasks were found in similar regions, namely right IPS, right MOG, and right/bilateral SFG. Activations in these regions are widely discussed in mental rotation studies and have been associated with visuo-spatial image transformation, working memory, motor simulation, motor planning/execution, and monitoring (Zacks, 2007; Arsalidou and Taylor, 2011; Fias et al., 2013). Because the Mental Rotation condition was chosen as a more complex visuo-spatial task, it is in line with our expectations that we found additional activation in frontal regions. The opposite contrasts (Numerical versus Mental Rotation, Perceptive Spatial versus Mental Rotation) revealed in both cases activation in the SFG, which has been associated with goal directed behavior (Arsalidou and Taylor, 2011). In the Numerical condition bilateral insula activation was found. This region has been implicated in various cognitive and affective functions, such as acting as an integral hub in the salience network (Menon and Uddin, 2010). In the literature on numerical and spatial cognition, the insula has mainly been associated with math anxiety (Lyons and Beilock, 2012) and time processing (Lewis and Miall, 2006), which are unlikely to play a role in discrete or continuous magnitude processing. Interestingly, a recent fMRI study investigating time, space and numerosity also found insula activation, suggesting that the insula plays a direct role in magnitude processing (Skagerlund et al., 2016). Regarding the fact, that our tasks are very similar to the ones used in Skagerlund et al. (2016) our data further supports the involvement of the insula in magnitude processing.

Taken together, differences in task specific activation is mainly explained by the extent to which domain-general functions are involved in the single conditions. On the other hand, similar activation patterns in the occipito-parietal lobe were observed in the tasks containing magnitude processing, indicating a comparable involvement of the domain-specific areas for the magnitude conditions. Hence, the results from our first research question would support the notion of a generalized magnitude system.

### DD Have Well Developed Abilities to Process Discrete and Continuous Magnitudes

In our second research question, we intended to investigate differences regarding the general magnitude system in adolescents with and without dyscalculia. If a shared magnitude system exists, it would be reasonable to find deficits in tasks containing spatial magnitude processing in adolescents with DD, taking into account their deficits in numerical processing. Alternatively, dissociation in these two abilities could possibly argue for partially overlapping or even separate systems.

Our DD group performed significantly worse than the control group in all numerical tasks, showing difficulties in basic arithmetical skills. According to our hypothesis, we further found significantly lower performances in a variety of behavioral visuo-spatial tasks. More precisely, DD participants performed significantly worse in the visuo-cognitive and visuo-constructive tasks, but reached similar levels in all visuo-perceptive tasks compared to the TD group. Several other studies have also reported deficits in visuo-spatial abilities in DD, although most of them examined only one of the various spatial components (Rourke and Finlayson, 1978; Osmon et al., 2006; Skagerlund and Träff, 2014). Yet, the observed deficits to date are mostly visuo-cognitive and visuo-constructive tasks (block design, judgment of line orientation, paper folding, and mental rotation), which is in line with our results. Our results are further supported by the finding that adults with DD seem to have well developed abilities to process continuous quantities, but show deficits in other numerical tasks (Cappelletti et al., 2014). Notably, accuracies in our behavioral length and size estimation task are near to ceiling levels and might therefore not disclose subtle differences between the groups. This seems unlikely, as no differences in the position estimation task and the Perceptive Spatial condition of the fMRI task could be found between the groups. However, as cognitive and constructive visuo-spatial tasks involve higher order functions (executive functions) to a greater extent, we cannot exclude the possibility that the significant IQ differences between the groups partially explain the lower performance in these tasks.

In the Numerical condition of the fMRI task, adolescents with DD did not show a deficient performance. Previous findings about non-symbolic magnitude processing are inconclusive (Price et al., 2007; Mussolin et al., 2010b; Piazza et al., 2010; Kucian et al., 2011b; Castro Cañizares et al., 2012; Landerl, 2013; Skagerlund and Träff, 2014). In addition, it has been shown that numerosity is not processed independently from its continuous visual variables (diameter, total surface, density; Gebuis and Reynvoet, 2012). Often the extent to which continuous visual properties of dot patterns are controlled in studies differs, which limits the comparability of the studies. In the present study, we controlled the continuous visual properties for our non-symbolic trials by first varying the size of dots in its diameter within the single items, second keeping the total surface constant between items, and third spreading the dots on a big or small area independent of their numerosity. Consequently, subjects were forced to mainly solve the task by a numerical decision. Regarding our data, we may therefore conclude, that adolescents with DD do not show a deficit in discrete non-symbolic comparisons. Furthermore, the results from both our groups are comparable to performance in adults as shown by Leibovich and Henik (2014). However, our data does not exclude the possibility, that deficits in non-symbolic magnitude processing are present at an earlier age in DD.

Regarding brain activation, only tasks containing magnitude decisions elicited neuronal differences between the groups (**Figure 4**). In both these tasks, subjects with DD showed increased activation in the left IFG (pars triangularis), an area known to be important for inhibition and updating information (Nee et al., 2013). More specifically, activation of the left IFG has strongly been associated with verbal content of working memory tasks and play an important role when relevant information has to be selected amid competition (Nee et al., 2013). On the other hand, the right IFG has been specifically associated with the spatial representation of numbers (Rusconi et al., 2011). Furthermore, a study investigating discrete and continuous magnitude representation in primates further showed that the IPS and the prefrontal cortex are involved in non-numerical magnitude representation (Tudusciuc and Nieder, 2009). This might suggest that dyscalculic participants rely more on domain-general abilities to solve the task (for instance higher need of updating information because of a deficit in magnitude processing), as often reported in the literature (e.g., Kaufmann et al., 2011; Kucian et al., 2011b). However, the activation in the IFG might also reveal some involvement of frontal magnitude processing areas. In contrast to the DD subjects, TD adolescents produced stronger activation in the left MOG, part of the dorsal extrastriate cortex, when processing continuous magnitudes. This area was associated in different studies with magnitude processing (Bueti and Walsh, 2009; Gijssels et al., 2013) indicating that TD adolescents use more task-specific regions to solve the task. Interestingly, no differences in the parietal lobe were found between groups, even when lowering the significance threshold to *p* < 0.005. On the one hand, this could reflect that adolescents with DD process simple numerosities and magnitudes in the same way as their peers. On the other hand, the more inferior areas of the occipital lobe might play a more important role in the approximate processing of numerosities than assumed. In fact, group differences were found in the MOG (MNI: *x* = -29, *y* = -86, *z* = 15) which has also been reported to be involved in rough estimations of quantity in the study of Roggeman et al. (2011; MNI: *x* = -25, *y* = -91, *z* = 3).

In summary, DD adolescents show in addition to the known deficits in numerical-arithmetical processing difficulties in cognitive and constructive visuo-spatial processing. Abilities to process non-symbolic (discrete) and perceptive visuo-spatial (continuous) magnitudes seem not to be affected in adolescents with DD. On the neuronal level, the increase in frontal activation might hint to the use of compensatory domain-general regions, revealing possible difficulties in dyscalculic adolescents. TD peers have increased activation in task relevant areas probably using a more efficient way of processing magnitudes. In this context, the deficits in number processing and arithmetic cannot be explained with a general magnitude deficit. This challenges the conclusion of previous studies that DD results from a deficit in the approximate magnitude representation (e.g., Piazza et al., 2010; Butterworth et al., 2011). Furthermore, adolescents with DD only show visuo-spatial deficits in more complex tasks which involve executive functions to a greater extent and do not contain magnitude processing. This better complies with the view that multiple neuro-cognitive components contribute to DD (Fias et al., 2013).

Leibovich and Henik (2013) suggest a developmental model with regard to a generalized magnitude system, which is in agreement with the present results. They argue that from an evolutionary point of view a “quick and dirty” estimation is sufficient. The first stage in development is the innate ability to discriminate continuous magnitudes. This is followed by learning the relationship between discrete and continuous properties: larger area and density usually means more numerous. In a further stage, children are able to integrate discrete and continuous properties to discriminate magnitudes. Lastly, with formal education symbolic representations of numbers are learned, allowing the detection of exact differences between magnitudes (Leibovich and Henik, 2013; see also von Aster and Shalev, 2007). According to our results, adolescents with DD seem to have accomplished the first three stages in the proposed developmental model, although they show some difficulties reaching the last stage compared to TD adolescents. Further studies are needed looking into typical as well as atypical development to confirm our results. We also propose to test the ability to process continuous and discrete magnitudes in younger children with DD to find out if these abilities are maintained or deficient at a younger age.

In summary, adolescents with DD seem to have a well-developed magnitude system for discrete and continuous sizes favoring the proposed theory of a generalized magnitude system (Walsh, 2003). To explain the additional difficulties in higher order visuo-spatial tasks and the substantial deficits in numerical and arithmetical skills requires more knowledge about the developmental trajectory of the magnitude system and the interactions with different cognitive domains. Finally, a multiple-component view might provide further insight into the pathophysiology of DD.

### Limitations

In the present study some limitations regarding group differences, paradigm design and interpretation of the data have to be taken into account.

Firstly, the revealed differences in the estimated IQ between adolescents with and without dyscalculia might limit the interpretation of the results. However, IQ tests are known to be not completely independent from numerical skills, and differences in IQ measures between a group of children with learning disabilities and a control group are often reported (Geary et al., 2000; Landerl, 2013; Willcutt et al., 2013). All our participants were well matched for comorbid measures and reached IQ scores in normal range, clearly fulfilling the diagnostic criteria for DD. In this context, the fact that we did not find differences in discrete and continuous magnitude processing, despite IQ differences, actually strengthens our findings. Furthermore, structural studies report deficient fiber projection between parietal, temporal and frontal regions in children with DD (Rykhlevskaia et al., 2009; Kucian et al., 2013). Intact white matter projections linking frontal and parietal areas seem to be crucial for performance in general intelligence (Gläscher et al., 2010; Kucian et al., 2014). This evidence could further explain why children with DD often score lower in IQ tests. In the context of the present arguments, we think that our dyscalculic participants represent the clinical population better than a population selected or artificially matched for IQ.

Secondly, although carefully planned and developed, the paradigm did not control for eye movements. This is important to consider, as studies show that saccades activate bilateral areas of the SPL and parts of the IPS (Simon et al., 2002, 2004; Culham and Valyear, 2006). We accounted for this problem by presenting our paradigm via video goggles, thus controlling the distance from the subject to the screen. Furthermore, the horizontal visual field of view was only 30°, minimizing eye movements to a small area. While presenting the same stimuli in all conditions, the subject had always to consider both items for a judgment of the task. We therefore assume that eye movements between conditions and subjects differ only slightly and do not affect the present results substantially. We propose for further studies to control eye movement by an eye tracking device.

Finally, our results point to the existence of a generalized magnitude system. However, it is important to note that in addition to providing a key region for number processing, the parietal lobe is reported to show activation in various tasks of spatial, motor, and attentional functions (Simon et al., 2002, 2004; Hubbard et al., 2005). We can therefore not exclude the possibility that the present results are partly based on overlapping attentional effects, as it is not yet known how attention and magnitude processing interact in the parietal lobe.

## Conclusion

The results obtained in the present study favor the possibility of a generalized magnitude system in the occipito-parietal lobe. It might be further assumed, that with development more refined and specific neuronal functions form in order to process magnitudes with increasing difficulty (Leibovich and Henik, 2013). Secondly, despite the numerical deficits and difficulties in more complex spatial skills, adolescents with DD seem to have well developed abilities to process discrete and continuous magnitudes. Neuronal findings may reveal the use of compensatory systems, hinting to a slight delay in the development of the discrete and continuous numerical system. Further studies are needed to examine the development of the generalized magnitude system in typical and, more importantly, in atypical development.

## Author Contributions

All authors have contributed and have approved the final manuscript. UM contributed to the design of the study, the acquisition, analysis and interpretation of the data, and writing the manuscript. MvA and RO contributed to data interpretation and revised the manuscript. KK contributed to the design of the study, data interpretation, editing and revising the manuscript.

## Conflict of Interest Statement

The authors declare that the research was conducted in the absence of any commercial or financial relationships that could be construed as a potential conflict of interest.
