# Supplementary material for: Adolescents with Developmental Dyscalculia Do Not Have a Generalized Magnitude Deficit – Processing of Discrete and Continuous Magnitudes
Source: Front Hum Neurosci. 2017 Mar 20;11:102. doi: 10.3389/fnhum.2017.00102 (PMC5357648; doi:10.3389/fnhum.2017.00102)
Supplement: Supplementary file 1 [file Data_Sheet_1.pdf]

Supplementary Material

Adolescents with Developmental Dyscalculia Do Not Have a Generalized Magnitude Deficit - Processing of Discrete and Continuous Magnitudes

Ursina McCaskey*, Michael von Aster, Ruth O'Gorman Tuura, Karin Kucian

*** Correspondence:** Ursina McCaskey: ursina.mccaskey@kispi.uzh.ch

1 Basic Diagnosis in Mathematics Education for Grades 4-8 (BASIS-MATH 4-8)

The BASIS-MATH test battery is a criterion-referenced test conceptualised to identify children with deficits in the basic mathematical concepts from grades 4 to 8. In accordance to the WHO definition of developmental dyscalculia, the mathematical performance is measured by the criterion "not reaching mastery of the basic mathematical concepts". The BASIS-MATH is composed of three difficulty levels measuring several arithmetical abilities. The subjects have to solve 48 tasks, which base on the arithmetic and mathematical knowledge acquired in grade 1 through 4. Each correct answer is rewarded with 1 to 2 points. The test has no time limit. Criteria for DD are met if the performance is under a threshold value of 67 points (maximum score 83 points).

The difficulty levels are set as follows: *Difficulty level I*: Skills in basic arithmetic operations in the number range 0-100. These skills correspond to the requirements of the second grade. *Difficulty level II*: Skills in basic arithmetic operations in the number range 0-1000. *Difficulty level III*: Skills in the place-value-system and understanding of the basic mathematical concepts.

The arithmetical abilities included in the test are:

- *Mental and written calculation*: Children have to solve additions, subtractions, multiplications and divisions of different difficulties (e.g., 47+36, 1000:8).
- *Subsets*: Children have to complete subsets and build part-whole-relationships (e.g., 1000 - ? = 670).
- *Counting*: The child is asked to count forward and backward in steps of two, ten and hundred (e.g., count backward in steps of ten starting at 137).
- *Decimal system*: Children have to create subsets, solve tasks regarding the place-value-system and make number line estimations (e.g., write down the number: 7 thousands, 3 tens and 15 units).
- *Problemsolving*: Three story problems have to be solved (e.g., 3 pieces of cheesecake cost CHF 7.20. How much does 1 piece cost?).

The BASIS-MATH is scaled according to the one-dimensional Rasch model. The normative sample consists of 692 Swiss and German children from grades 4 to 8. The test has a high sensitivity (92%) and reliability (internal consistency Cronbach's alpha = .92).

2 Conjunction analyses

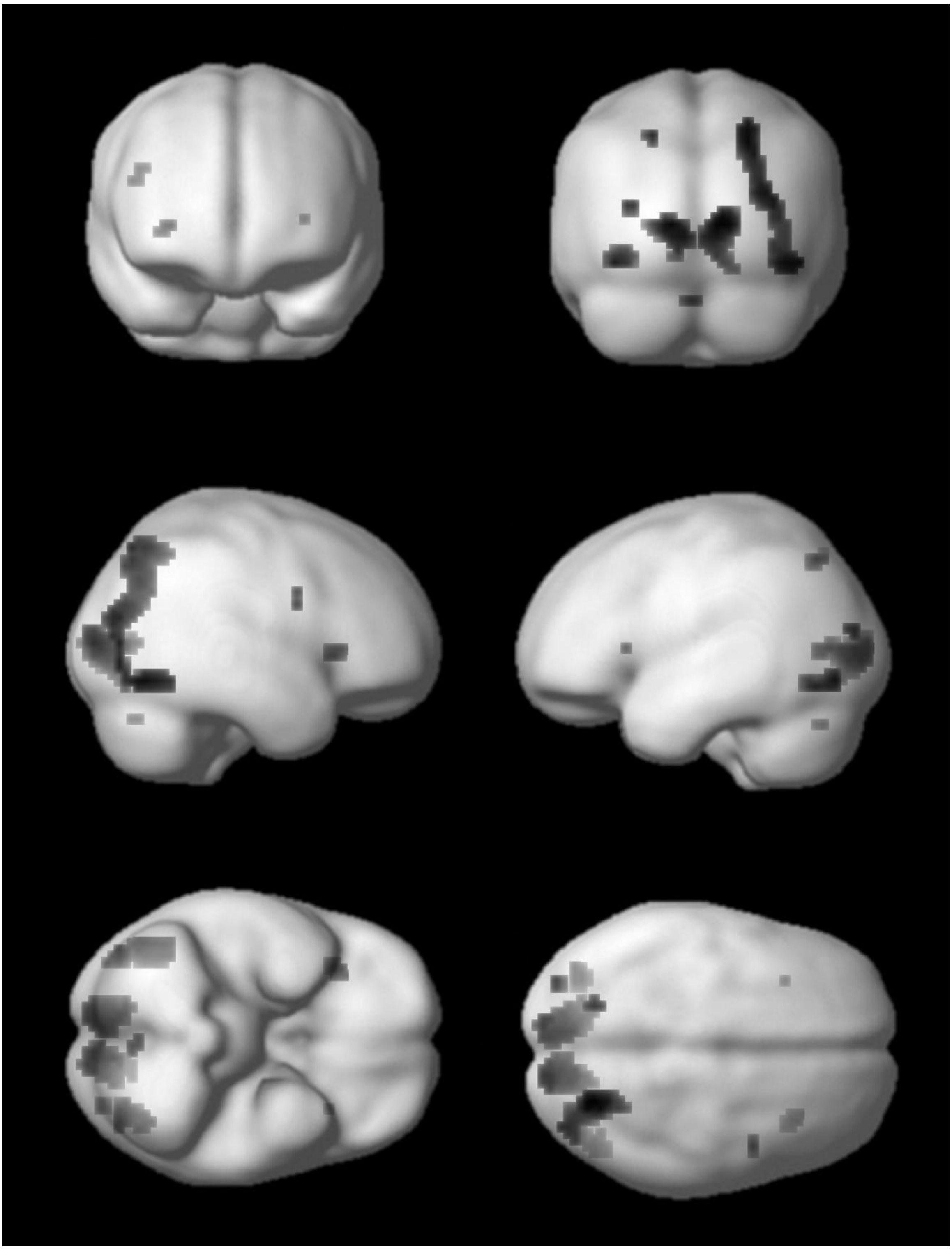

Figure S1. Commonly activated regions for all experimental conditions: Numerical, Perceptive Spatial and Mental Rotation (experimental vs. control; $p < 0.05$, $k \geq 5$, FWE corrected).

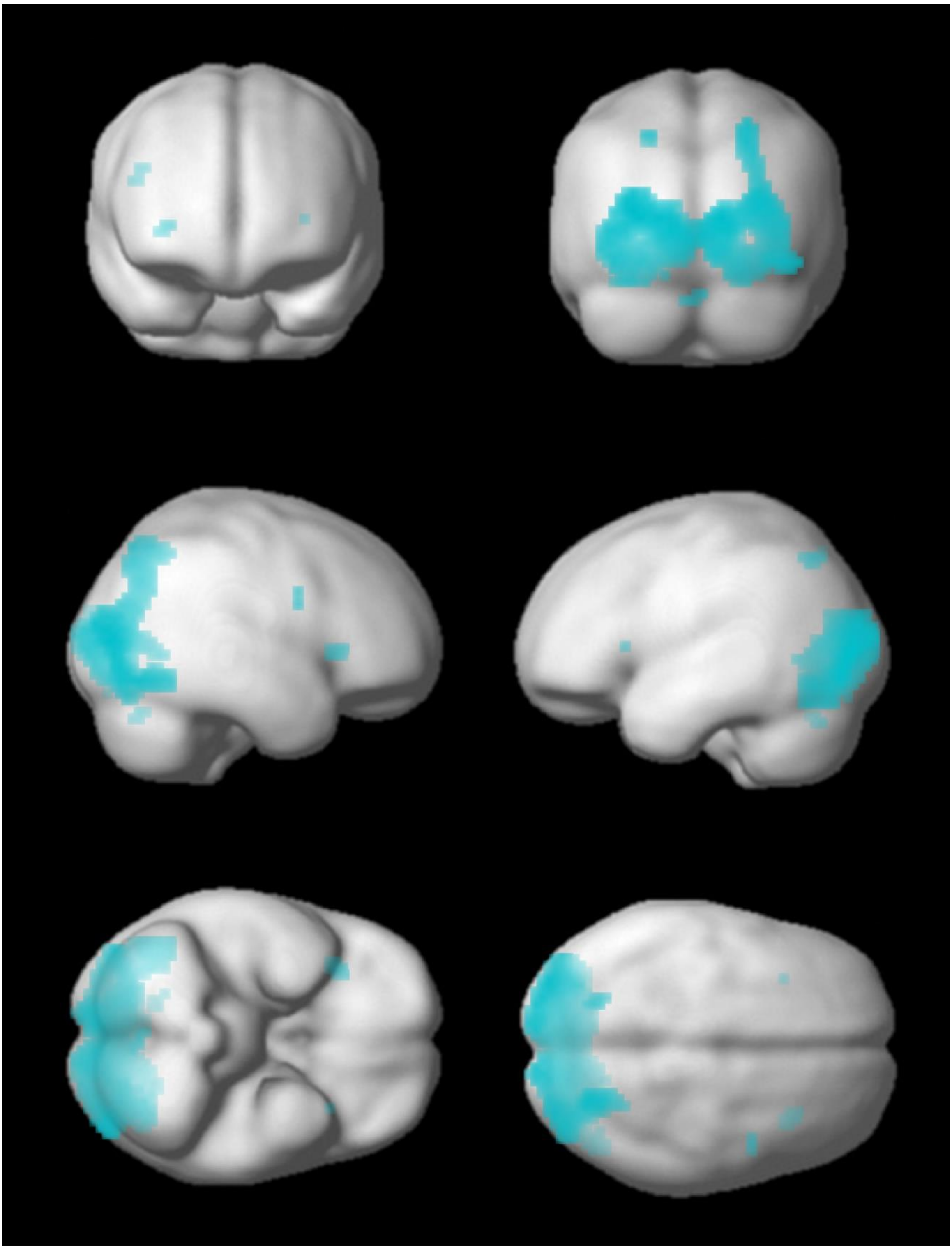

Figure S2. Commonly activated regions for the magnitude conditions: Numerical and Perceptive Spatial (experimental vs. control; $p < 0.05$, $k \geq 5$, FWE corrected).

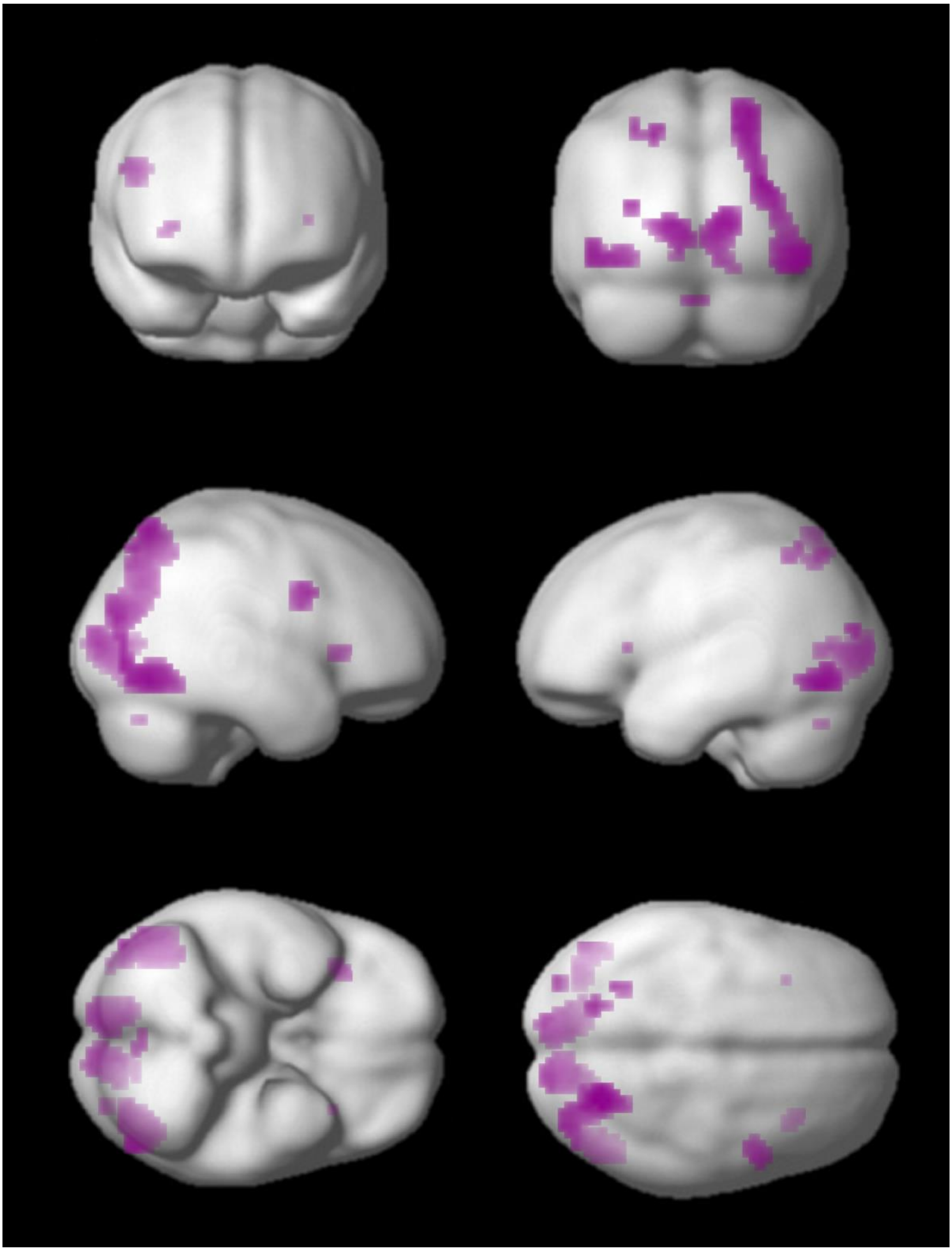

Figure S3. Commonly activated regions for the spatial conditions: Perceptive Spatial and Mental Rotation (experimental vs. control; $p < 0.05$, $k \geq 5$, FWE corrected).

3 Conjunction analyses for the DD and the TD group

Conjunction null analyses for the magnitude conditions ([Numerical > Control] ∩ [Perceptive Spatial > Control]) were conducted for the DD and the TD group separately.

Results show that for both groups similar regions are commonly activated for the tasks containing magnitude processing (Figure S4 and Table S1). However, the extent of the commonly activated regions in the DD group is much smaller compared to the TD group. Furthermore, activation reaching into the right superior occipital lobe cannot be found in the DD group. This difference might be explained by the higher inter-individual variability found in DD subject (Kucian et al., 2006) or underlying differences in magnitude processing between groups.

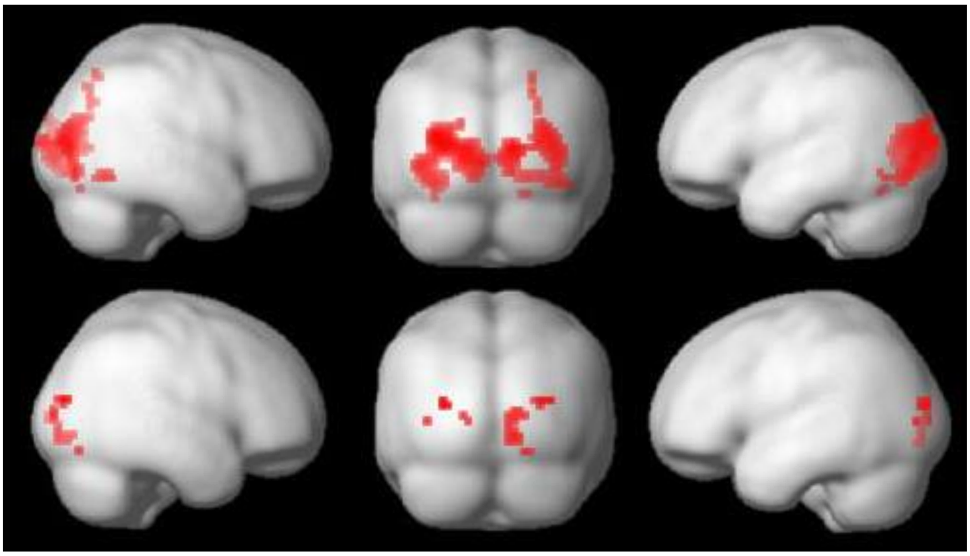

Figure S4. Commonly activated regions for the magnitude conditions (Numerical and Perceptive Spatial condition) in the TD (upper row) and the DD group (lower row) ($p < 0.05$, k ≥ 5, FWE corrected).

Table S1. Brain areas that showed significant activation in the conjunction analyses for the magnitude conditions in the TD and the DD group ($p < .05$, k ≥ 5, FWE corrected).

**Region**	**Cluster size**	**Peak t-value**	**Peak MNI coordinates**		
			**x**	**y**	**z**
**Conjunction analyses magnitude conditions**					
Typically Developing					
L middle occipital gyrus	499	8.59	-29	-89	15
L inferior occipital gyrus		6.75	-32	-80	-9
L superior occipital gyrus		6.47	-14	-86	6
R calcarine gyrus	288	6.51	13	-83	6
R middle occipital gyrus		6.14	31	-80	18
R inferior occipital gyrus	18	5.31	43	-62	-12
R superior occipital gyrus	7	4.93	28	-71	36
Developmental Dyscalculia					
R calcarine gyrus	19	5.09	16	-92	6
R middle occipital gyrus	8	5.33	31	-86	15
L middle occipital gyrus	6	5.19	-26	-89	12
